# Healthcare Debt Among Older Adults With Low-Income: Evidence From a Mixed-Methods

**DOI:** 10.1093/geroni/igaf122.3952

**Published:** 2025-12-31

**Authors:** Anam Bhatti, Ivan Avila, Hannah Beesley, Jeffrey Greger

**Affiliations:** AARP Foundation, Washington, District of Columbia, United States; AARP Foundation, Washington, District of Columbia, United States; AARP Foundation, Washington, District of Columbia, United States; AARP Foundation, Washington, District of Columbia, United States

## Abstract

Although Medicare and Medicaid extend broad coverage, some older adults with low-income (OALI) still experience healthcare debt, underscoring ongoing vulnerabilities in financial and health security. The AARP Foundation conducted 12 focus groups (n = 81), 13 expert interviews, and a nationally representative survey (n = 2,066) of adults aged 50+ with low-income. Qualitative data analysis primarily followed a grounded theory approach. Nationally weighted survey data were examined via cross-tabulations to assess debt prevalence, drivers, and consequences by income, insurance type, age, and comorbidity status. Using a broader definition of healthcare debt than benchmark studies (e.g., SIPP and KFF), this study finds that coverage gaps and provider misperceptions about financial aid create lasting financial burdens for OALI. Survey findings show that 33% of Medicare and 39% of Medicaid beneficiaries carry healthcare debt, most often linked to dental, and vision services. Qualitative themes reinforce survey findings: charity care screening targets uninsured OALI yet falls short for those on high-deductible plans, contributing to low awareness (26%) and use (16%). Debt forces tradeoffs: 44% of OALI cut back on basic needs and 45% of those owing over $1,000 delay or forgo treatment altogether. The study findings underscore the need to boost assistance awareness and tailor interventions by income and coverage type. The findings offer insights for stakeholders to design financial assistance programs that prevent debt accumulation and safeguard both the health and financial security of older adults.

